# A New Method for the Determination of Sucrose Concentration in a Pure and Impure System: Spectrophotometric Method

**DOI:** 10.1155/2017/8214120

**Published:** 2017-12-28

**Authors:** A. Borji, Fz. Borji, A. Jourani

**Affiliations:** Laboratoire PCPM, Faculté des Sciences et Techniques, Université Hassan 1er, BP 557, Settat, Morocco

## Abstract

Analytical chemistry is a set of procedures and techniques used to identify and quantify the composition of a sample of material. It is also focused on improvements in experimental design and the creation of new measurement tools. Analytical chemistry has broad applications to forensics, medicine, science, and engineering. The objective of this study is to develop a new method of sucrose dosage using a spectrophotometry method in a pure and impure system (presence of glucose and fructose). The work performed shows the reliability of this method. A model linking sucrose solution absorbance and mass percentage of glucose and fructose has been developed using experimental design. The results obtained show that all the investigated factors (sucrose concentration, mass percentage of glucose, and mass percentage of fructose) have a positive effect on the absorbance. The effect of the interaction between glucose and fructose on the absorbance is very significant.

## 1. Introduction

Analytical chemistry is a measurement science consisting of a set of powerful ideas and methods that are useful in all fields of science and medicine. It is applied throughout industry, medicine, and all the sciences [1].

Dosage has a primary role in the field of chemical analysis, allowing the user of a product or a substance quick and precise answers to any question related to its characterization.

Sucrose and other carbohydrates can be easily distinguished, either by taste or by means of easily developed physical and chemical reactions. The dosage of the latter can be achieved by several methods, either by physical, chemical, or biological methods using enzymes [2, 3].

Dosage by physical methods is used to dose sucrose in technical or purified sugar solutions, especially those which are marketed under the name of “liquid sugars”. These techniques will mainly be used for high-sucrose processed products [4]. The best known methods are polarimetry [5–7], refractometry [8, 9], and those using densimeter.

In spite of their high degree of accuracy, physical methods can only be used for the titration of pure sucrose solutions. If solutions are more complex, with the presence of reducing sugars, colorants, or flavor enhancers, chemical methods can be employed to determine sucrose content. The most widely used chemical methods are chromatography [10, 11] and reducing sugars, which are based on the reducing properties of free carbonyl group in reducing sugars, reacted with a copper solution in alkaline medium [12].

As for biological methods, their principle is extensively described in the specialized literature, especially in Bergmeyer's work [13].

The objective of this paper is to develop a new method for the determination of sucrose by spectrophotometer. The influence of certain impurities, such as glucose and fructose, on the sucrose dosage, using this method, has been studied.

## 2. Materials and Methods 

### 2.1. Reagents

The reagents used in this study are

- sucrose;
- glucose;
- fructose.

The reagents employed are “of analytical” quality to avoid any other impurities which may influence the measurements.

### 2.2. Preparation of Samples

The solutions prepared for sucrose dosage are

- stock solution of 1.9 mg/mL sucrose;
- diluted solutions of concentration: respectively, 0.304, 0.646, 0.95, 1.33, and 1.9 g/mL of sucrose.

### 2.3. Experimental Protocol

The absorbance of all diluted samples from the stock solution was measured using a spectrophotometer at a wavelength of 420 *μ*m. The measurements of the refractive index have been performed in order to compare between spectrophotometric and refractometric methods.

## 3. Experimental Results

### 3.1. Dosage of Sucrose

Among the most frequently used methods for sucrose dosage in aqueous solutions, we find the refractometric method. The bijective relation between the refractive index of pure sucrose solution and its concentration can be used to titrate sucrose in aqueous solutions [14]. For this reason it was chosen as a tool for comparison with the spectrophotometric method.

**Figure 1:**
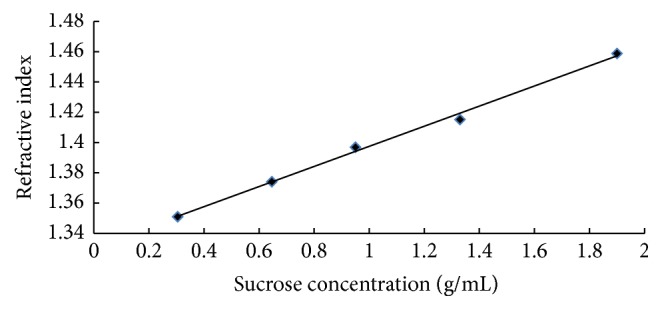
Calibration curve for the refractometer.

**Figure 2:**
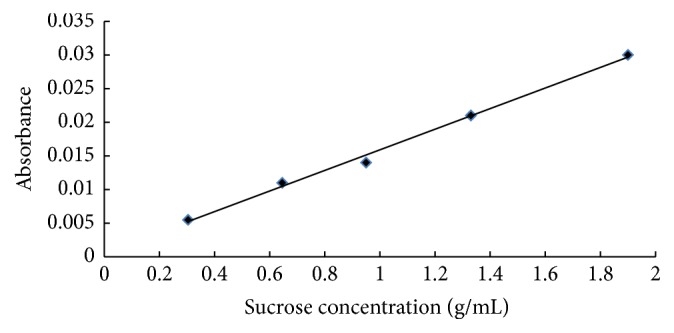
Calibration curve for the spectrophotometer.

The calibration equations for the refractometer and the spectrophotometer obtained are, respectively, shown in Figures 1 and 2.

These results indicate that there is good correlation between sucrose concentration and absorbance on the one hand and refractive index on the other, whose calibration equations are Ab = 0.0153*C* + 0.0006 for the spectrophotometer, with a correlation coefficient of 99.5%, and *n* = 0.0664*C* + 1.3311 for the refractometer, with a correlation coefficient of 99.6%, where Ab, *n*, and *C* are the absorbance, the refractive index, and the sucrose concentration, respectively.

Repeated tests (4 times) confirm the reproducibility of these results, with the standard deviations being 10^−4^ and 8.33 × 10^−4^ for spectrophotometer and refractometer, respectively.

In order to test these two methods, the absorbance and the refractive index of known concentrations of sucrose solutions were measured. The results obtained have been compared with those calculated by the calibration equations (see Tables 1 and 2).

These results show the reliability of sucrose dosage by spectrophotometric method. It should be noted that the refractometric method is more sensitive than the latter. The sensitivity of these two methods, with respect to glucose and fructose, has been studied.

### 3.2. Influence of Glucose

**Figure 3:**
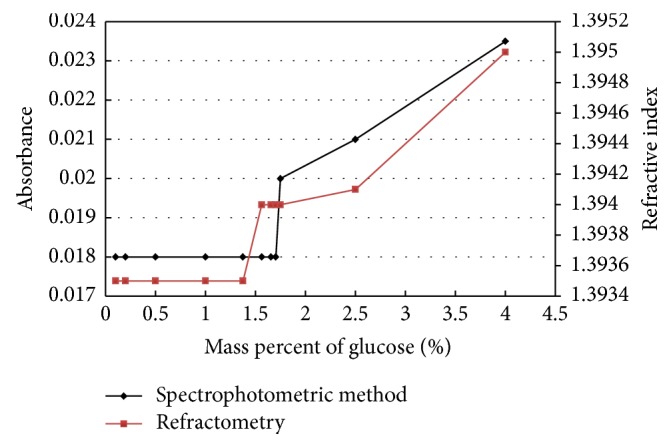
Influence of glucose on absorbance and refractive index.

After the sucrose dosage in aqueous solutions by spectrophotometric method has been validated, the influence of glucose on the absorbance and on the refractive index has been studied.  Figure 3 shows the variation of the absorbance and the refractive index as a function of the mass percentage of glucose and for a sucrose concentration of 0.5 g/mL.

From these results (Figure 3), it can be seen that the effect of glucose starts from 1.56% for the refractometer and from 1.75% for the spectrophotometer. Therefore, the latter can be used to titrate sucrose solutions in impure system (presence of glucose with a percentage which does not exceed 1.75%). In the following, the absorbance will be modeled as a function of the sucrose concentration and the mass percentages of glucose and fructose, using the experimental design, with the aim of developing a tool capable of measuring the sucrose concentration in impure system (presence of glucose and fructose).

### 3.3. Modeling of Sucrose Solution Absorbance

Effect of glucose and fructose on the sucrose solution absorbance was studied, using the experimental design. A model linking the sucrose solution absorbance and these two monosaccharides has been established.

The technique of statistical design for the experiments can be used for process characterization, optimization, and modeling.

Basically, the classical parameter design is complicated and not easy to use; in particular, a large number of experiments must be conducted when the number of the process parameters increased. For this reason, the design of experiments is a useful tool to study the interactions between two or more variables at reduced number of experimental trials [15]. The factorial designs determine which factors have the important effects on the response and how the effect of one factor varies with the level of the other factors. The effects are the differential quantities expressing how a response changes as the levels of one or more factors are changed. Also, factorial designs allow measuring the interaction between each different group of factors [16].

If we call *n* the number of variables to be tested, in order to measure the effect of all the variables combinations when each variable is tested at a high and a low level, 2^n^ experiments will be needed [17]. In this study, three factors were chosen as independent variables, namely, concentration of sucrose (*x*_1_), mass percent of glucose (*x*_2_), and mass percent of fructose (*x*_3_).

The natural values of each factor and their respective levels are presented in Table 3. The selection of levels of different factors is carried out on the basis of the preliminary trials: sucrose concentration ranging from 0.3 to 1.1 g/mL, mass percent of glucose from 0.1 to 4 %weight, and mass percent of fructose from 0.1 to 4 %weight. The design performed according to Table 4 was composed of 2^3^ factorial designs.

Table 4 shows the results of the 8 tests carried out.

The coded values of *x*_*j*_ were obtained from the following relationship [18–20]:

(1) $$\begin{array}{c} x_{j}=\frac{Z_{j}-Z_{j}^{0}}{\Delta Z_{j}}\;j=1,2,\ldots ,k, \end{array}$$

with

(2) Zj0=Zjmax+Zjmin2,ΔZj=Zjmax−Zjmin2,

where *x*_*j*_ is the coded value of *j*th variable, *Z*_*j*_ is the encoded value of *j*th variable, *Z*_*j*_^0^ is the value of *Z*_*j*_ at the center point of the investigation domain, and Δ*Z*_*j*_ is the step size. Here, *Z*_*j*max_ and *Z*_*j*min_ represent the maximum and the minimum level of factor *j* in natural unit, respectively. The experimental data are analyzed by full factorial design to fit the following first-order polynomial equation [21–23]:

(3) y=b0+ε+b1x1+b2x2+b3x3+b12x1x2+b13x1x3+b23x2x3+b123x1x2x3,

where *y* the estimated sucrose solution absorbance; *b*_0_ is the value of fitted response at the center point of design; *b*_*j*_ and *b*_*ji*_ are the linear and interaction terms, respectively [24, 25].

When the response data are obtained from the test work, a regression analysis is carried out to determine the coefficients of the response model (*b*_1_; *b*_2_; …; *b*_*n*_), as well as their standard errors and their significance. In addition to the constant (*b*_0_) and error (*ε*) terms, the response model incorporates [26]

- linear terms in each of the variables (*x*_1_, *x*_2_,…, *x*_*n*_);
- first-order interaction terms for each paired combination (*x*_1_*x*_2_, *x*_1_*x*_2_,…, *x*_*n*−*i*_*x*_*n*_).

In general (3) can be written in matrix form:

(4) $$\begin{array}{c} Y=BX+\varepsilon . \end{array}$$

The *B* coefficients, which should be determined in the second-order model, are obtained by Goupy [21]:

(5) $$\begin{array}{c} B={\left[\;X^{T}\cdot X\;\right]}^{-1}\cdot {\left[\;X\;\right]}^{T}\cdot Y, \end{array}$$

where *B* is the column matrix of estimated coefficients; [*X*^*T*^ · *X*]^−1^ the dispersion matrix; [*X*]^*T*^ the transpose matrix of experiments matrix [*X*] and *Y* is the column matrix of observations.

The model equation for absorbance of sucrose solution was obtained after performing eight experiments and discarding the insignificant effect (*b*_13_ and *b*_123_):

(6) y^=0.02500+0.008000x1+0.002500x2+0.002750x3−0.001000x1x2+0.001750x2x3.

The model's coefficients were estimated using Minitab software.

In order to validate this model, it is tested using the center, 1/4, and 3/4 of the range of each factor. The results obtained are summarized in Table 5.

According to this test, we find that the empirical model gives results closer to reality, so it can be used to measure sucrose, either in pure or in impure system (presence of glucose and fructose). The difference between the measured and the predicted values does not exceed 0.1%.

The values obtained by the model ($\hat{y}$ predicted) are compared with those of experimental data (*y* experimental) (Table 6).

**Figure 4:**
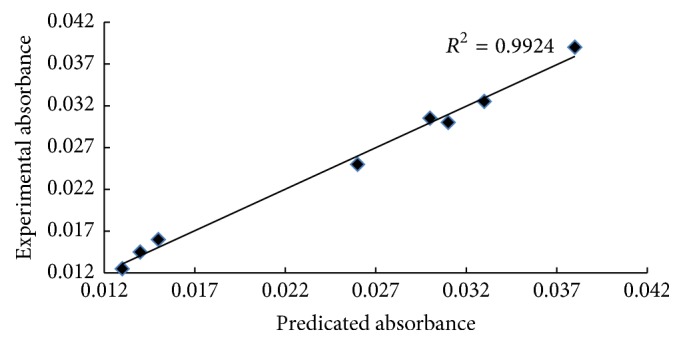
Comparison of experimental and predicted responses.

**Figure 5:**
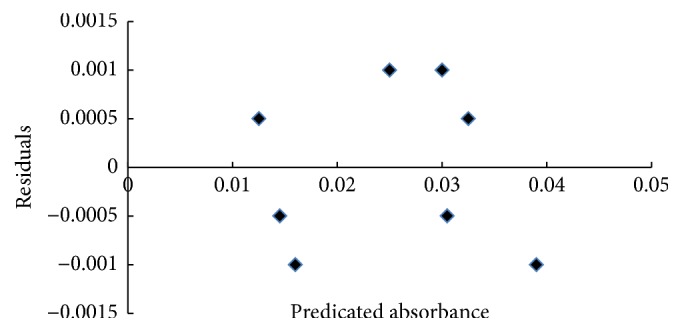
Residual analysis for estimated model.

A good adjustment of (6) to the experimental data was verified through the high correlation coefficient value obtained: *R*^2^ = 99.24% (Figure 4). The random distribution of the residuals (Figure 5) shows the absence of a trend, indicating that the mathematical model is adequate and that there is no inconsistency between the experimental and calculated values of the response.

**Figure 6:**
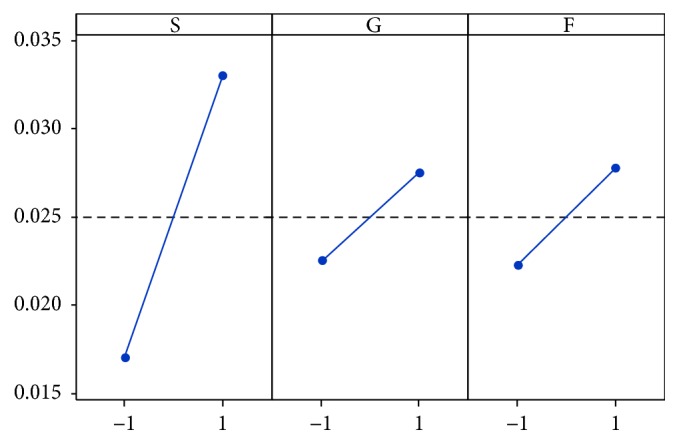
Diagram of the main effects for absorbance (S: sucrose; G: glucose; F: fructose).

**Figure 7:**
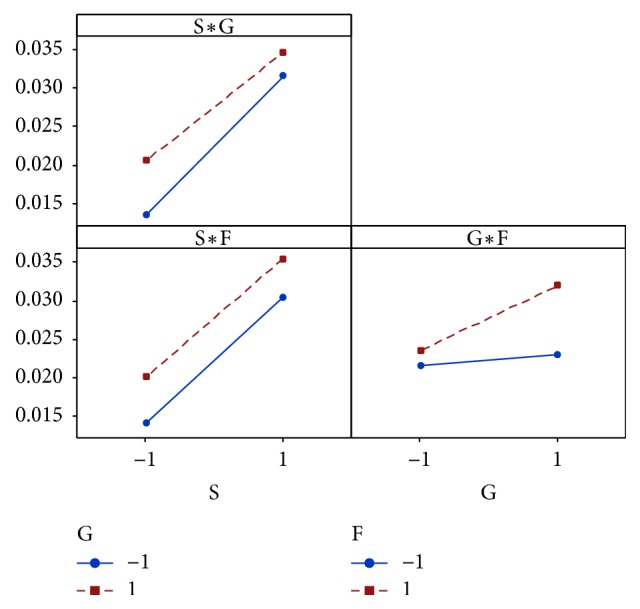
Diagram of interactions for absorbance (S: sucrose; G: glucose; F: fructose).

Figures 6 and 7 illustrate the effects of each factor and their interactions on the sucrose solutions absorbance.

The results show that increasing the percentage of glucose and fructose causes an absorbance increase. The effects of glucose-sucrose and fructose-sucrose interactions are negligible because the effect of these tow monosaccharides on absorbance does not depend on sucrose concentration. On the contrary, the effect of the interaction between fructose and glucose is very significant.

## 4. Conclusion

In this work, a new spectrophotometric method for the determination of sucrose concentration in a pure and impure system was demonstrated. The calibration equation established was validated and compared with the refractometric method. An empirical model linking the absorbance of sucrose solution and mass percentage of glucose and fructose was developed using the experimental design.

The work performed shows the reliability of this method and that glucose and fructose have a positive effect on the sucrose solution absorbance. Influence of the interaction between these two monosaccharides on absorbance is very significant.

## Figures and Tables

**Table 1:** Validation of the calibration equation for the spectrophotometric method.

Concentration (g/mL)	0.418
Absorbance	0.008
Calculated concentration	0.438
Absolute error	0.020

**Table 2:** Validation of the calibration equation for the refractometer.

Concentration (g/mL)	1.267
Refractive index	1.416
Calculated concentration	1.279
Absolute error	0.012

**Table 3:** The experimental ranges and levels of independent variables.

Factors	Symbol	Low level (−1)	High level (+1)	Unit
Concentration of sucrose	*x*_1_	0.3	1.1	g/mL
Mass percent of glucose	*x*_2_	0.1	4	% weight
Mass percent of fructose	*x*_3_	0.1	4	% weight

**Table 4:** Experimental design matrix.

Experiment	*I*	*x*_1_	*x*_2_	*x*_3_	*y*
(1)	1	−1	−1	−1	0.013
(2)	1	1	−1	−1	0.030
(3)	1	1	1	−1	0.031
(4)	1	−1	−1	1	0.014
(5)	1	1	1	1	0.038
(6)	1	−1	1	−1	0.015
(7)	1	−1	1	1	0.026
(8)	1	1	−1	1	0.033

**Table 5:** Comparison between the measured absorbance and the calculated absorbance.

Test	Calculated absorbance	Measured absorbance	Absolute error
(0,0, 0)	0.0250	0.026	0.001
(0.5,0.5,0.5)	0.0317	0.032	0.0003438
(−0.5, −0.5, −0.5)	0.0186	0.019	0.0004063

**Table 6:** Comparison between observed and predicted responses.

Runs	*y*	$\hat{y}$	Residuals
(1)	0.013	0.0125	0.0005
(2)	0.03	0.0305	−0.0005
(3)	0.031	0.03	0.001
(4)	0.014	0.0145	−0.0005
(5)	0.038	0.039	−0.001
(6)	0.015	0.016	−0.001
(7)	0.026	0.025	0.001
(8)	0.033	0.0325	0.0005
